# The Effect of General Anesthesia vs. Regional Anesthesia on Postoperative Delirium—A Systematic Review and Meta-Analysis

**DOI:** 10.3389/fmed.2022.844371

**Published:** 2022-03-28

**Authors:** Xianlin Zhu, Min Yang, Junying Mu, Zaiping Wang, Liang Zhang, Hongbai Wang, Fuxia Yan

**Affiliations:** ^1^Department of Anesthesiology, The Central Hospital of Enshi Tujia and Miao Autonomous Prefecture, Enshi City, China; ^2^Department of Anesthesiology, Chongqing Hospital of Traditional Chinese Medicine, Chongqing, China; ^3^Department of Anesthesiology, Fuwai Hospital, Chinese Academy of Medical Sciences and Peking Union Medical College, Beijing, China

**Keywords:** general anesthesia, regional anesthesia, neuraxial anesthesia, peripheral nerve block, postoperative delirium, meta-analysis

## Abstract

**Background:**

Postoperative delirium (POD) commonly occurs in patients following major surgeries and is associated with adverse prognosis. The modes of anesthesia may be associated with POD occurrence. General anesthesia (GA) causes loss of consciousness in the patient by altering the levels of some neurotransmitters as well as signaling pathways. We conducted this meta-analysis to investigate the effect of GA vs. regional anesthesia (RA) on POD incidence in surgical patients.

**Methods:**

The databases of Pubmed, Embase, and Cochrane Library were searched till October 22, 2021. The eligible criteria were participants aged 18 years or older, patients undergoing surgery under GA and RA, and articles reporting the effect of GA vs. RA on POD incidence. RevMan 5.3 was used to perform statistical analyses.

**Results:**

A total of 21 relevant trials with a total of 1,702,151 patients were included. The pooled result using random-effects model with OR demonstrated significant difference in POD incidence between patients with GA and RA (OR = 1.15, 95% CI: [1.02, 1.31], *I*^2^ = 83%, *p* for effect = 0.02). We did not obtain the consistent pooled result after sensitivity analysis (OR = 0.95, 95% CI: [0.83, 1.08], *I*^2^ =13%, *p* for effect = 0.44) and excluded the articles without the information on preoperative cognitive or neuropsychological assessment (OR = 1.12, 95% CI: [1.00, 1.25], *I*^2^ =80%, *p* for effect = 0.05), respectively.

**Conclusion:**

This meta-analysis could not identify that GA was significantly associated with POD occurrence in surgical patients compared with RA.

## Introduction

Postoperative delirium (POD) is a kind of acute complication characterized by brain dysfunction following surgery, and its main manifestations include inattention, disorganized thinking, and altered states of consciousness (1). Commonly, POD occurs in the first 3 days following surgery, and its higher incidence is in patients undergoing cardiac and major non-cardiac surgeries (2, 3). Furthermore, POD is independently associated with other short- and long-term postoperative complications, adversely affecting prognosis (4–6). Given that there is no effective treatment for POD due to its unclear pathogenesis, identifying its risk factors is particularly important (7). Previous studies have identified some risk factors of POD, including major surgery, advanced age, lower educational level, preoperative anxiety, perioperative poor sleep quality, and imperfect postoperative analgesia (8).

Anesthesia is a critical intervention process for surgical patients. There are several methods of anesthesia, like general anesthesia (GA) and regional anesthesia (RA). RA includes neuraxial anesthesia (epidural anesthesia or spinal anesthesia) and peripheral nerve block (PNB). GA is the anesthesia method that keeps the patient in the state of unconsciousness, analgesia, and relaxed skeletal muscle through intravenous and/or inhaled general anesthetics during surgery. Some anesthetics act on the central nervous system to produce the effects of consciousness loss and analgesia by altering the levels of some neurotransmitters as well as signaling pathways (9, 10). However, the neuraxial anesthesia and PNB can contribute to analgesia and skeletal muscle relaxation without affecting the consciousness of the patient. Therefore, the patients undergoing GA are theoretically more likely to develop POD. Unexpectedly, the results of anesthesia mode associated with POD incidence are not consistent based on previous studies (11). Thus, we performed this systematic review and meta-analysis to clarify the effect of GA vs. RA on the incidence of delirium in adult patients undergoing surgery.

## Methods

This systematic review and meta-analysis were conducted according to the guidelines of the 2009 Preferred Reporting Items for Systematic Reviews and Meta-Analyses (PRISMA) (Supplementary Table 1) (12).

### Search Strategy

Two authors independently searched the database of Pubmed, Embase, and Cochrane Library using the PICOS (Population, Intervention, Comparison, Outcome, and Study design) methods by the time of October 22, 2021. The entry terms included “general anesthesia” OR “general anesthesia” AND “local anesthesia” OR “local anesthesia” OR “regional anesthesia” OR “regional anesthesia” OR “spinal anesthesia” OR “spinal anesthesia” “epidural anesthesia” OR “epidural anesthesia” OR “neuraxial anesthesia” OR “neuraxial anesthesia” AND “delirium” OR “confusion” OR “agitation” OR “acute confusional state” OR “acute confusional syndrome,” and the search field was “title and abstract.” Since we sought to investigate all studies about the effect of GA vs. RA on POD incidence in adult patients undergoing surgery, we did not constrain the search terms for study designs.

### Study Selection

Two authors were independently responsible for the screening process for titles and abstracts, while another two authors conducted the screening process for full text. The inclusion criteria were: (1) participants aged 18 years or older, (2) patients undergoing surgery under general and regional or local anesthesia, and (3) articles reporting the effect of GA vs. RA on POD incidence. The exclusion criteria were: (1) duplicate articles, (2) participants younger than 18 years old, (3) review or meta-analysis, (4) articles published as an abstract, letter, case report, basic research, editorial, note, method, or protocol, (5) articles presented in a non-English language; (6) studies without a specific number of patients with and/or without delirium, and (7) studies of all patients receiving GA or RA.

### Quality Assessment of Included Studies

Two authors independently assessed the quality of included studies. For retrospective and prospective observational trials, the risk of bias was assessed using the Newcastle–Ottawa Quality Assessment Scale (NOS), which comprises the following three domains: selection, comparability, and outcome for cohort studies (13). There were four stars in the selection domain, two stars in the comparability domain, and three stars in the exposure domain. Trials with seven or more cumulative stars were considered to be of high quality, those with six stars of moderate quality, and those with <6 stars of low quality (13). For RCTs, risk of bias was assessed using the Cochrane Collaboration Risk of Bias Assessment tool, which included the following seven items: random sequence generation, allocation concealment, blinding of participants and personnel, blinding of outcome assessment, incomplete outcome data, selective reporting, and others (bias due to vested financial interest and academic bias) (14). If a trial was found to have one or more of the items associated with a high or unclear risk of bias, this trial was classified as high risk. If the two authors disagreed on their assessment, they consulted the third or fourth author. Eventually, we reached a consensus (14).

### Data Extraction

Two authors were responsible for extracting the following information: (1) authors, (2) publication year, (3) study designs, (4) country of publication, (5) total number of participants in each study, (6) percentage of male, (7) mean age of all the participants, (8) procedures that the participants underwent, (9) the volatile anesthetic in patients underwent GA, (10) the anesthetic method of RA, (11) number of patients with and without POD, (12) methods of POD assessment, and (13) the follow-up time. Another three authors were responsible for adjusting data discrepancies.

### Outcome Measures

The sole aim of this meta-analysis was to determine whether different anesthesia methods were associated with POD incidence in adult patients undergoing surgery.

### Data Analysis

Review Manager version (RevMan) 5.3 (Cochrane collaboration, Oxford, UK) was used to perform statistical analyses. We assessed the heterogeneity of included studies using the values of *I*^2^ and the Mantel–Haenszel chi-square test (*p*-value for heterogeneity). The values of *I*^2^ <40%, *I*^2^ = 40–60%, and *I*^2^ > 60% indicated low, moderate, and high heterogeneity, respectively (15). If we identified *I*^2^ > 50% or a *p*-value for heterogeneity <0.1, we used a random-effect model to analyze the data. Conversely, if we identified *I*^2^ <50% or a *p*-value for heterogeneity ≥ 0.1, we used a fixed-effect model to analyze the data (16). The dichotomous outcomes were presented as odds ratios (OR) with 95% confidence intervals (CI). The statistical tests were two-sided, and overall effects with a *p*-value for effect < 0.05 were considered to exhibit significant differences.

We conducted a sensitivity analysis to address high heterogeneity (*I*^2^ > 50%) through the methods of one-by-one article removal. Lastly, we performed the subgroup analyses according to study designs (retrospective and prospective), male percentage (≥50 and <50%), age gaps (≥80, 70–80, 60–70, and <60 years), and anesthesia methods (neuraxial anesthesia and PNB) in RA group to observe if these risk factors could affect the outcome.

## Results

### Study Selection

**Figure 1:**
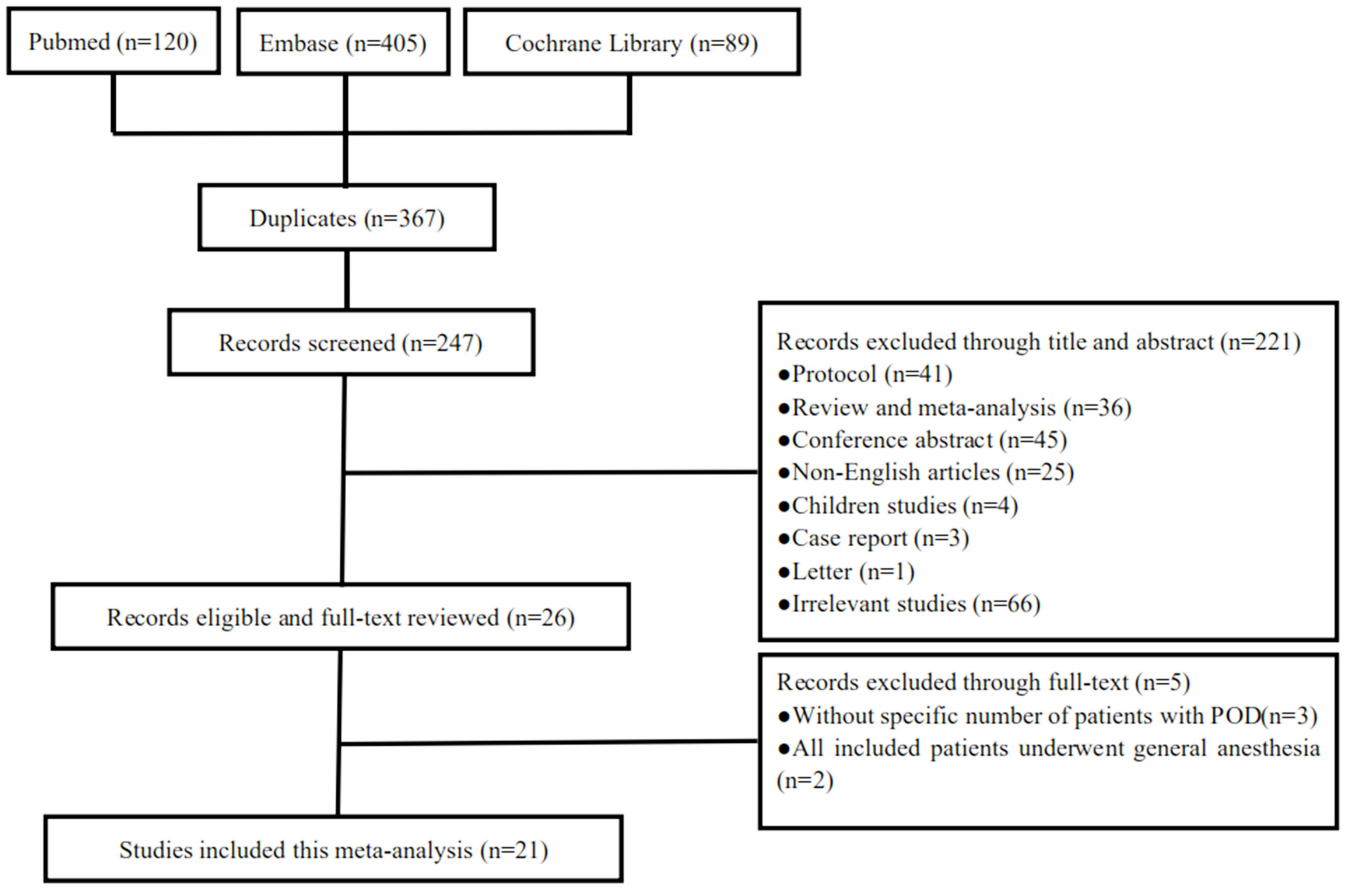
The screening process of the eligible trials.

Figure 1 shows the PRISMA flow chart for our screening process. We obtained 120 trials from Pubmed, 405 from Embase, and 89 from Cochrane Library. We removed 367 duplicate trials and excluded 221 trials at the title-and-abstract review stage based on our exclusion criteria. We excluded five trials at the full-text review stage, including three without a specific number of patients with POD, two with all included patients who underwent general anesthesia. Eventually, our search strategy yielded 21 relevant trials with a total of 1,702,151 patients (Figure 1) (17–37).

### Study Characteristics

Tables 1, 2 present the basic characteristics of the included studies. There were nine retrospective (17, 18, 22, 25, 28–31, 35), and 12 prospective (19–24, 26, 27, 32–34, 36, 37) articles, including seven randomized and non-randomized controlled trials, in the prospective studies (20, 23, 24, 32–34, 37). Male accounted for 50% or more of all patients in 4 trials (17, 22, 23, 25). The mean or median age of all included patients was 80 years or older in five trials (20, 26, 31–33), 70–80 years in seven trials (21, 28, 29, 34–37), 60–70 years in seven trials (17, 18, 23–25, 27, 30), and younger than 60 years in two trials (19, 22). Except that the patients underwent lower limb vascular surgery in two articles (23, 25) and uncertain surgeries in one article (19), the patients underwent orthopedic surgery in the other trials. One trial included the patients undergoing simple urgent surgery (29), and five trials simple planned surgery (19, 21, 24, 27, 30). Three articles did not provide information on preoperative cognitive or neuropsychological assessment (23, 28, 35). The patients in the RA group received the sole PNB in only two trials (17, 29), and underwent PNB or neuraxial anesthesia in another two trials (25, 35), while neuraxial anesthesia was the sole anesthesia in RA patients in the other 17 enrolled trials. The follow-up time was hospital stay after surgery in 13 trials (18, 22, 25–33, 35, 36), 1 year in one trial (23), 30 days in one trial (17), 1–7 days in one trial (20), 1–5 day in one trial (34), 1–4 days in one trial (37), 1–3 days in two trials (21, 24), and 1 day in one trial (19). The methods of POD identification included antipsychotic drug use (17, 18, 22, 30), Confusion Assessment Method for Intensive Care Unit (CAM-ICU) or CAM (19, 21, 36, 37), DSM-IV criteria (24, 27), NEECHAM confusion scale (25), change in mental state (23), and Pittsburgh Agitation Scale (26). Seven articles did not provide a specific diagnostic method of POD (20, 28, 29, 31–33, 35), and a combination of CAM with DSM-IV criteria (34).

**Table 1:** The basic characteristics of included trials.

**References**	**Study design**	**Country**	**Number of patients**	**Male (%)**	**Age (mean or median) (years)**	**Procedures**	**Urgent/Planned**	**Preoperative cognition assessment**	**RA**	**POD assessment**
Abe et al. (17)	Retrospective	Japan	11,796	69.1	69.2	Lower extremity amputation	Both	Yes	PNB	Based on newly prescribed antipsychotic drugs
Ahn et al. (18)	Retrospective	Korea	96,289	25.7	79	Hip surgery	Both	Yes	Neuraxial anesthesia	Based on administration of antipsychotic drugs
Bilge et al. (19)	Prospective	Turkey	250	43.2	59.7	Operation planned with general and regional anesthesia	Planned	Yes	Regional anesthesia	CAM-ICU
Casati et al. (20)	Prospective (RCT)	Italy	30	6.7	84	Hip surgery	Both	Yes	Neuraxial anesthesia	NA
Chew et al. (21)	Prospective	Singapore	462	29.9	72	Knee or hip surgery	Planned	Yes	Neuraxial anesthesia	CAM
Choi et al. (20)	Retrospective	Korea	24,379	61.0	52.9	Hip surgery	Both	Yes	Neuraxial anesthesia	Diagnosis codes or administration of antipsychotic drugs
Cook et al. (23)	Prospective (RCT)	Australia	101	70	66.8	Lower limb vascular surgery	Both	No	Neuraxial anesthesia	Based on change in mental state
Ehsani et al. (24)	Prospective (controlled trial)	Iran	94	47.9	67.12	Hip surgery	Planned	Yes	Neuraxial anesthesia	DSM-IV
Ellard et al. (25)	Retrospective	Canada	500	68.4	69	Vascular surgery	Both	Yes	Neuraxial anesthesia or PNB	NEECHAM confusion scale
Ilango et al. (26)	Prospective	Australia	318	30.0	81.6	Hip surgery	Both	Yes	Neuraxial anesthesia	Pittsburgh Agitation Scale
Krenk et al. (27)	Prospective	Denmark	225	49.3	69.4	Total knee or hip arthroplasty	Planned	Yes	Neuraxial anesthesia	DSM-IV
Li et al. (28)	Retrospective	China	89	48.3	76.8	Lower lumbar surgery	Both	No	Neuraxial anesthesia	NA
Liu et al. (29)	Retrospective	China	217	30.4	79.8	Hip surgery	Urgent	Yes	PNB	NA
Memtsoudis et al. (30)	Retrospective	USA	169,4795	39.7	67.3	Total knee or hip arthroplasty	Planned	Yes	Neuraxial anesthesia	ICD-9 codes and/or billing for antipsychotics
Nawi et al. (31)	Retrospective	Australia	154	33.1	83.1	Hip surgery	Both	Yes	Neuraxial anesthesia	NA
Parker et al. (32)	Prospective (RCT)	UK	322	29.2	82.95	Hip surgery	Both	Yes	Neuraxial anesthesia	NA
Shin et al. (33)	Prospective (RCT)	Korea	176	26.1	80.5	Hip surgery	Both	Yes	Neuraxial anesthesia	NA
Slor et al. (34)	Prospective (controlled trial)	Netherlands	526	22.1	77.45	Hip surgery	Both	Yes	Neuraxial anesthesia	DSM-IV and CAM
Song et al. (35)	Retrospective	Korea	3,611	49.4	>70	Orthopedic surgery	Both	No	Neuraxial anesthesia or PNB	NA
Song et al. (36)	Prospective	China	138	26.1	78.35	Hip surgery	Both	Yes	Neuraxial anesthesia	CAM
Tzimas et al. (37)	Prospective (RCT)	Greece	70	47.1	76	Hip surgery	Both	Yes	Neuraxial anesthesia	CAM

*GA, general anesthesia; RA, regional anesthesia; POD, postoperative delirium; NA, not applicable; PNB, peripheral nerve block; CAM-ICU, the Confusion Assessment Method for Intensive Care Unit; RCT, randomized controlled trial; DSM-IV, the Diagnostic and Statistical Manual of Mental Disorders 4th Edition; ICD, International Classification of Diseases*.

**Table 2:** The follow-up time and number of patients with POD under different anesthesia modes.

**Author and year**	**Assessment time**	**GA**		**RA**	
		**With POD**	**Without POD**	**With POD**	**Without POD**
Abe et al. (17)	Within 30 days after surgery	1,017	10,032	69	678
Ahn et al. (18)	Hospital stay after surgery	5,828	19,765	12,733	57,963
Bilge et al. (19)	Postoperative 1 day	37	187	9	17
Casati et al. (20)	Postoperative 1–7 days	9	6	8	7
Chew et al. (21)	Postoperative 1–3 days	0	164	0	298
Choi et al. (22)	Hospital stay after surgery	142	9,779	209	14,249
Cook et al. (23)	1 year after surgery	6	45	9	41
Ehsani et al. (24)	Postoperative 1–3 days	14	33	2	45
Ellard et al. (25)	Hospital stay after surgery	73	323	24	80
Ilango et al. (26)	Hospital stay after surgery	84	83	88	63
Krenk et al. (27)	Hospital stay after surgery	0	22	0	203
Li et al. (28)	Hospital stay after surgery	4	38	0	47
Liu et al. (29)	Hospital stay after surgery	15	57	23	122
Memtsoudis et al. (30)	Hospital stay after surgery	28,933	974,263	13,579	545,573
Nawi et al. (31)	Hospital stay after surgery	46	112	10	42
Parker et al. (32)	Hospital stay after surgery	0	164	3	155
Shin et al. (33)	Hospital stay after surgery	17	101	8	50
Slor et al. (34)	Postoperative 1–5 days	18	42	171	295
Song et al. (35)	Hospital stay after surgery	165	2,373	7	1,066
Song et al. (36)	Hospital stay after surgery	24	57	12	45
Tzimas et al. (37)	Postoperative 1–4 days	4	29	10	27

### Study Quality

We used NOS to assess the risk of bias in observational studies (retrospective and prospective), and all 14 trials obtained seven stars or more, indicating high quality (Supplementary Table 2) (17–19, 21, 22, 25–31, 35, 36). We used the Cochrane Collaboration Risk of Bias Assessment tool to assess the risk of bias in RCTs. Due to the considerable procedural difference in GA and neuraxial anesthesia or PNB, it is extremely difficult to conduct blindness in participants. Therefore, the performance bias was high risk in all included RCTs and non-RCTs. The included studies clearly assessed random sequence generation (three studies-42.9%), allocation concealment (four studies-57.1%), blinding of participants (0%), blinding of outcome assessment (seven studies-100%), incomplete outcome data (seven studies-100%), and selective outcome reporting (seven studies-100%), and the other bias (three-42.9%) (Supplementary Figures 1, 2) (20, 23, 24, 32–34, 37).

### POD Incidence

**Figure 2:**
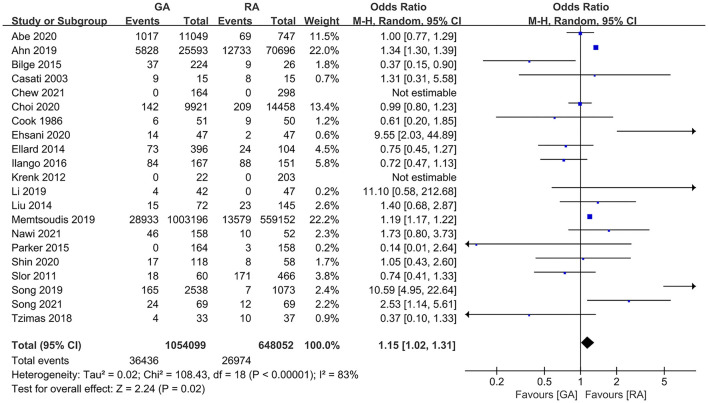
The pooled results of POD incidence after surgery between the patients with GA and RA.

**Figure 3:**
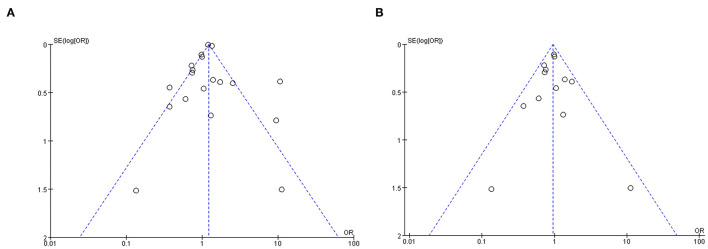
The funnel plots of all included trials: the considerable heterogeneity of included trials **(A)** and lowering heterogeneity through six trials exclusion **(B)**.

**Figure 4:**
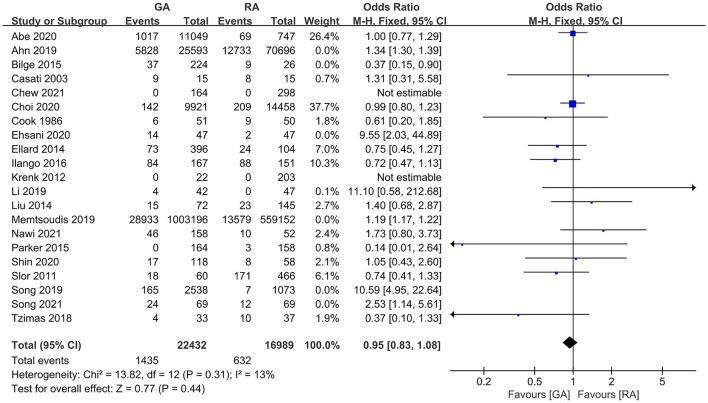
The pooled result of POD incidence in surgical patients with GA and RA after sensitivity analysis.

**Figure 5:**
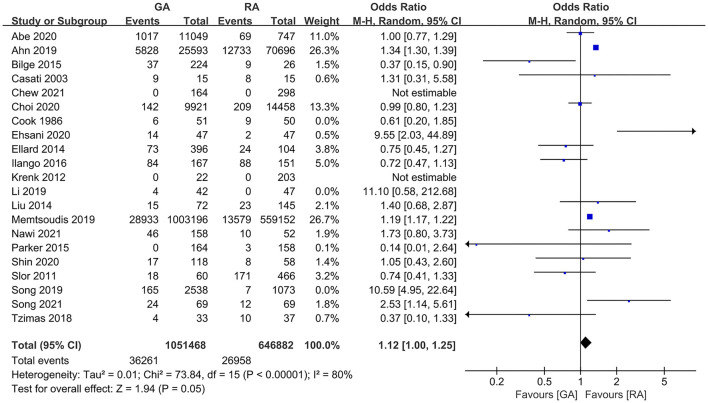
The pooled result of POD incidence in surgical patients with GA and RA after excluding the studies without the information on preoperative cognitive or neuropsychological assessment.

The pooled result using random-effects model with OR demonstrated significant difference in POD incidence between patients with GA and RA (OR = 1.15, 95% CI: [1.02, 1.31], *I*^2^ = 83%, *p* for effect = 0.02) (Figure 2). *I*^2^ = 81% and the funnel plots demonstrated the considerable heterogeneity of included trials (Figure 3A). The sensitivity analysis was performed to solve the high heterogeneity by the method of one-by-one literature removal and found that six trials were the main sources of heterogeneity (Figure 3B) (18, 19, 24, 30, 35, 36). We conducted *post-hoc* meta-analysis for the remaining articles using a fixed-effects model with OR, and the pooled result was not consistent with that prior to sensitivity analysis (OR = 0.95, 95% CI: [0.83, 1.08], *I*^2^ = 13%, *p* for effect = 0.44) (Figure 4). Additionally, we excluded the articles without the information on preoperative cognitive or neuropsychological assessment and did not obtain the statistical difference in POD incidence between patients with GA and RA (OR = 1.12, 95% CI: [1.00, 1.25], *I*^2^ = 80%, *p* for effect = 0.05) (Figure 5).

**Figure 6:**
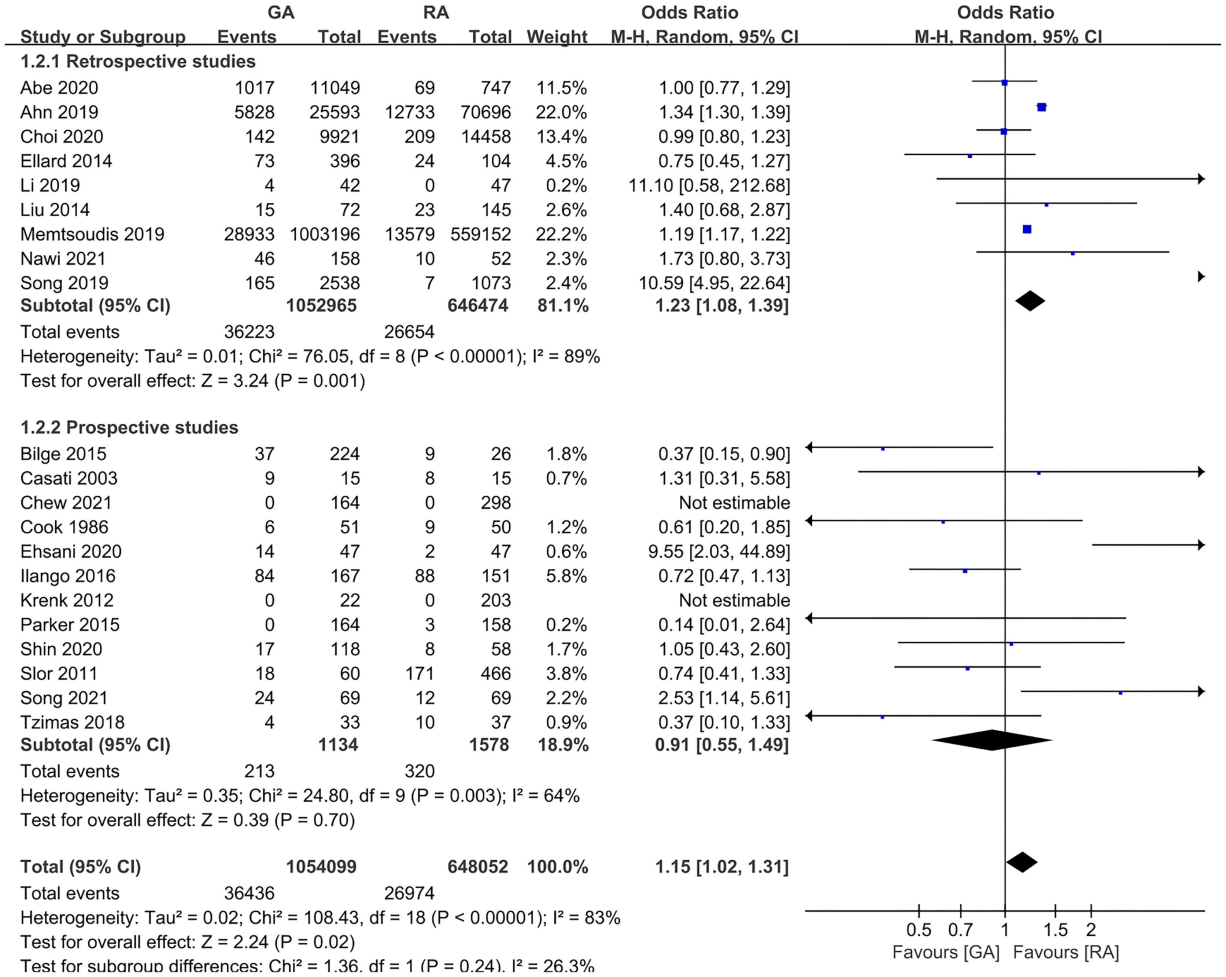
The subgroup analysis according to study designs (retrospective and prospective).

**Figure 7:**
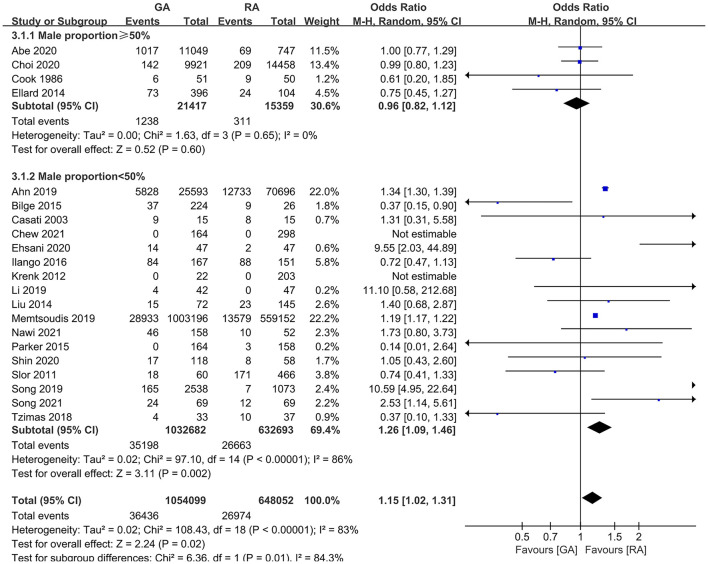
The subgroup analysis according to the male percentage (≥50 and <50%).

**Figure 8:**
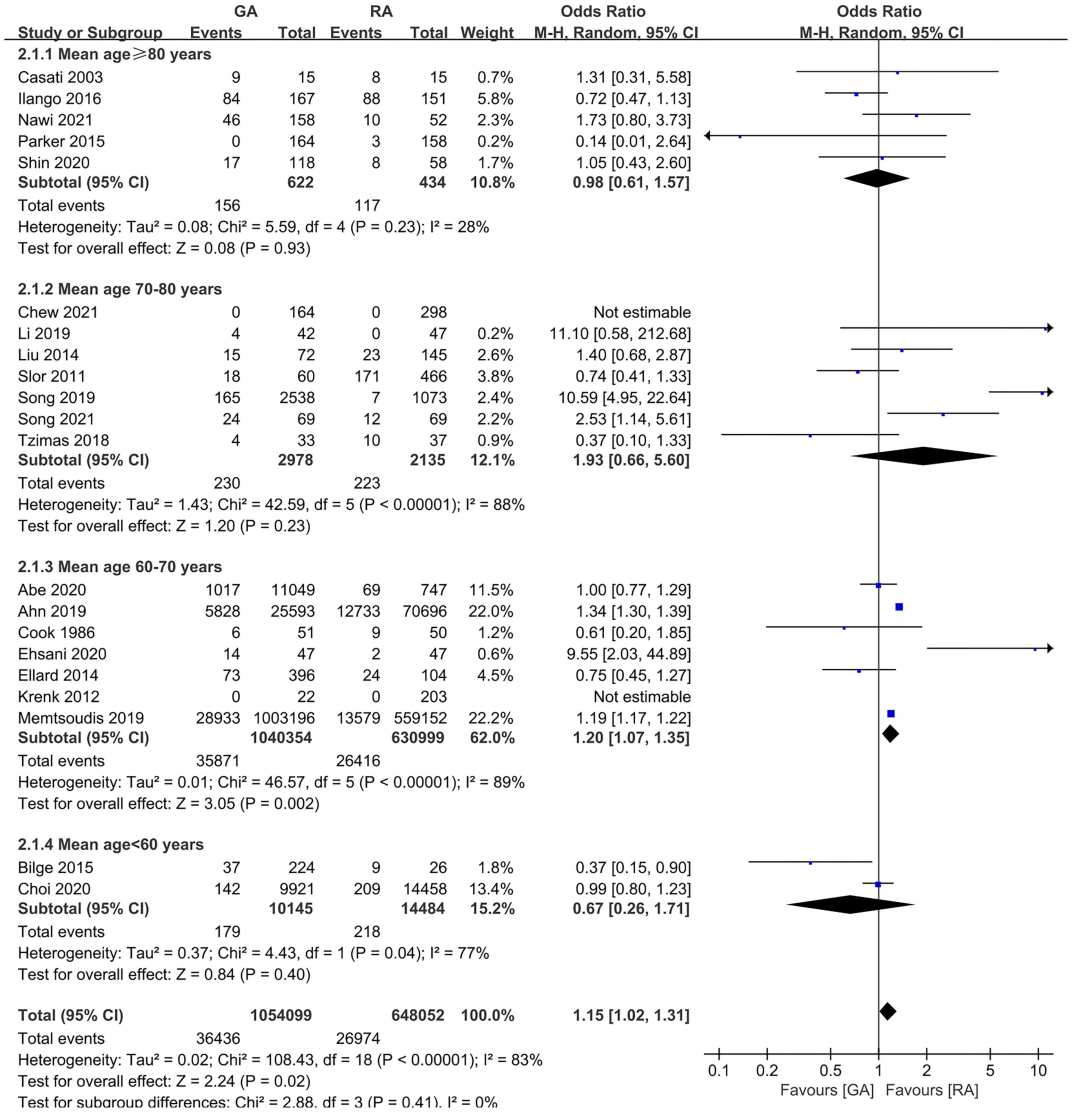
The subgroup analysis according to mean (or median) age gaps (≥80 years, 70–80 years, 60–70 years, and <60 years).

**Figure 9:**
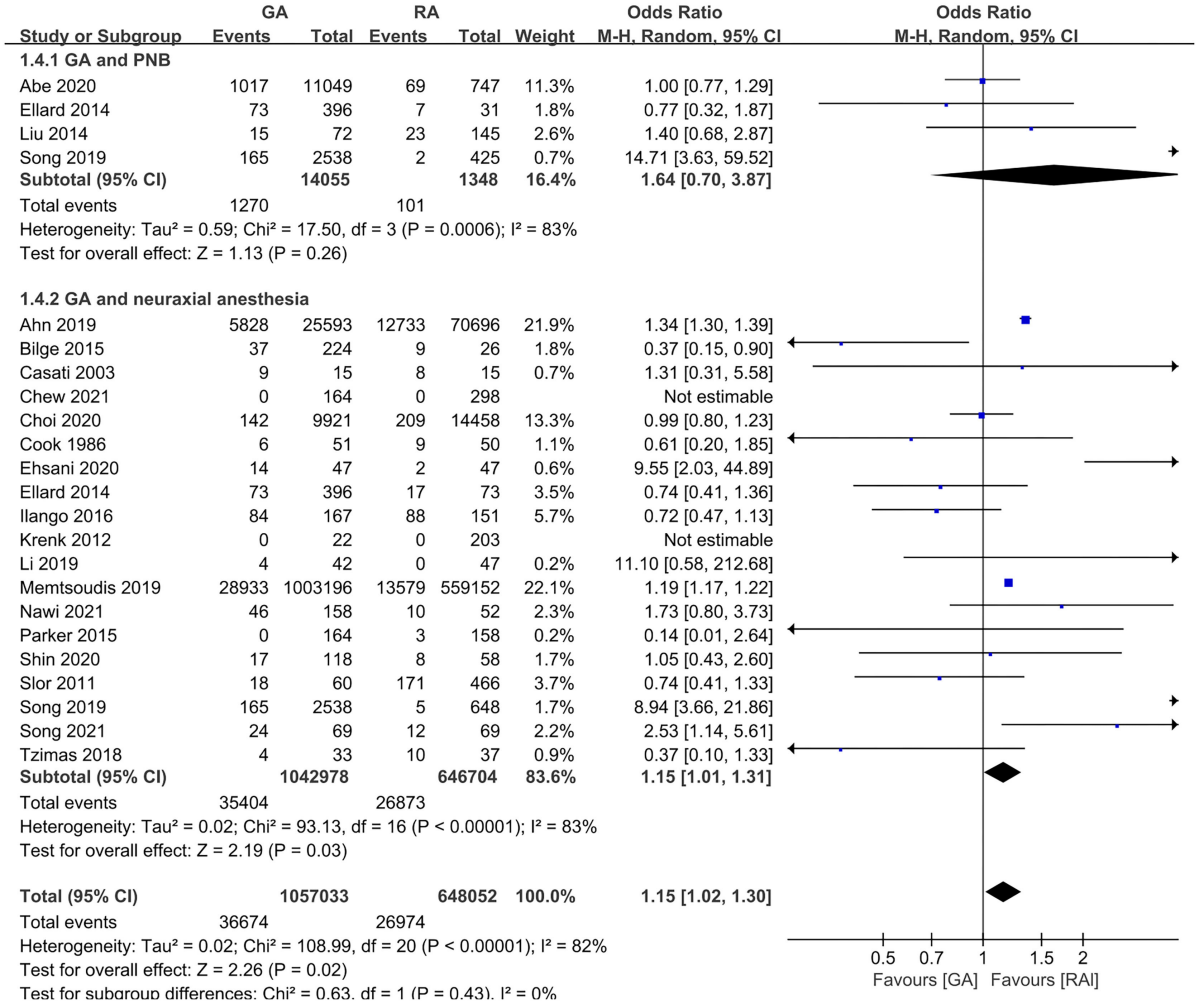
The subgroup analysis according to anesthesia methods in the RA group (neuraxial anesthesia and PNB).

The subgroup analyses according to study designs, male percentage (≥50 and <50%), mean (or median) age gaps (≥80, 70–80, 60–70, and <60 years), and anesthesia methods in RA group (neuraxial anesthesia and PNB) demonstrated the significant difference in retrospective articles (OR = 1.23, 95% CI: [1.08, 1.39], *p* for effect = 0.001) (Figure 6), male percentage <50% (OR = 1.26, 95% CI: [1.09, 1.46], *p* for effect = 0.002) (Figure 7), age gap between 60 and 70 years (OR = 1.20, 95% CI: [1.07, 1.35], *p* for effect = 0.002) (Figure 8), and neuraxial anesthesia (OR = 1.15, 95% CI: [1.01, 1.31], *p* for effect = 0.03) (Figure 9). However, we did not obtain statistical difference in the subgroups in prospective studies (OR = 0.91, 95% CI: [0.55, 1.49], *p* for effect = 0.70) (Figure 6), male percentage ≥ 50% (OR = 0.96, 95% CI: [0.82, 1.12], *p* for effect = 0.60) (Figure 7), age gaps ≥ 80 years (OR = 0.98, 95% CI: [0.61, 1.57], *p* for effect = 0.93), 70–80 years (OR = 1.93, 95% CI: [0.66, 5.60], *p* for effect = 0.23), <60 years (OR = 0.67, 95% CI: [0.26, 1.71], *p* for effect = 0.40) (Figure 8), and PNB group (OR = 1.64, 95% CI: [0.70, 3.87], *p* for effect = 0.26) (Figure 9).

## Discussion

Although this meta-analysis concluded that compared with RA, the incidence of POD significantly increased in GA patients, we did not obtain the positive result after solving the high heterogeneity of included trials and excluded the trials that did not provide the information on preoperative cognitive or neuropsychological assessment, respectively. Besides, subgroup analyses showed the statistical difference in retrospective studies, studies with male percentage <50%, studies with a mean (or median) age gap 60–70 years, and studies with neuraxial anesthesia group in RA group. However, we did not obtain the considerable difference in POD occurrence between the patients with GA and RA in the trials of prospective designs, the male percentage ≥ 50%, patients with another mean (or median) age gaps except 60–70 years, and patients undergoing PNB in the RA group.

Although the mechanisms of POD occurrence are complex and currently unclear, some studies have exhibited its possible pathogeneses. Severe neuroinflammation may be a main cause of POD in patients undergoing cardiac or non-cardiac surgery (38, 39). The serum S100A2 is a pro-inflammatory factor associated with POD and also a biomarker indicating neural injury according to a clinical study, and maybe an effective predictor of POD (40). Increasing perioperative plasma cortical level is considered as another mechanism of POD occurrence due to its related neuron apoptosis in the hippocampal region (41, 42). Besides, plasma neurofilament light level is also a predictor of POD, independent of changes in inflammation. Elevated plasma neurofilament light level is correlated with reduced hippocampal volume and fractional anisotropy of white matter (43). Furthermore, the preoperative neurotransmitter imbalances occurred in POD patients, such as increased dopamine and glutamate, and decreased glutamine, which potentially increase the fragility of the brain (44). Due to the complex and unclear mechanism of POD, currently, the main method to decrease POD incidence is the intervention of its perioperative risk factors, like preoperative fasting, temperature control, blood pressure management, perioperative sleep improvement, moderate anesthesia depth, and perfect analgesia (7).

General anesthesia can affect the individual conscious state through complex molecular biological mechanisms, including ligand-gated ionotropic receptors, like γ-aminobutyric acid, glutamate, and acetylcholine receptors, and then intervene synaptic transmission between neurons (45). However, the specific mechanism of action of general anesthetics is still elusive. According to animal and clinical studies, GA-related varieties of consciousness and cognition are reversible and transient (46, 47). But some studies exhibited that GA or general anesthetics could produce neural toxicities, and be associated with short- or long-term cognitive dysfunction, and the extent of cognitive defect was proportional to the duration of anesthesia (48, 49). The volatile anesthetics may be a critical risk factor of neural injury through elevating the neural injury biomarkers total tau, neurofilament light, and tau phosphorylation (50, 51). Additionally, GA can increase frontal slow-wave activity, and impaired functional connectivity on diffusion tensor imaging, which may be associated with POD occurrence (52). Besides, GA may disturb the postoperative sleep structure of patients, thereby resulting in POD (53). Therefore, more patients undergoing GA theoretically tend to develop POD. Interestingly, compared with RA, the patients receiving GA did not show significant POD incidence according to some studies (11).

In this meta-analysis, although the pooled result of all included trials demonstrated that POD incidence was higher in surgical patients undergoing GA than RA, the consistent result did not be obtained after solving the high heterogenicity of included trials. Besides, the pooled result from retrospective studies was positive, while the prospective ones were negative. Given that the retrospective studies have a higher incidence of selection and recall biases, we are not yet sure whether GA is associated with higher POD incidence than RA (54). Additionally, preoperative cognitive function or neuropsychological state of patients considerably affects their POD incidence. In this meta-analysis, all trials provided the cognitive or neuropsychological baseline information without statistical difference between GA and RA groups except for three articles (23, 28, 35). We excluded the three trials and found that GA did not significantly increase the POD incidence of patients compared with RA.

Subgroup analyses of this meta-analysis also exhibited unexpected results. According to previous studies, both male gender and advanced age are risk factors of POD occurrence (55, 56). Interestingly, in this meta-analysis, the pooled result of trials with a higher male percentage (≥50%) was not significant in POD incidence between patients with GA and RA, meanwhile, the advanced age did not exhibit a considerable difference in POD occurrence in patients between GA and RA, either. We consider the possible reasons, including the following ones: (1) The POD occurrence in female gender may be more susceptible to the modes of anesthesia; (2) the number of included trials in subgroup of male percentage ≥50% is too small to prove the result; (3) the patients aged 60–70 years are more affected by anesthesia modes in POD occurrence; (4) the subgroup of mean (or median) age gap of 60–70 years included the retrospective studies with large sample size of patients, while the sample size of patients in other mean (or median) age gaps is relatively smaller, respectively. Besides, we obtained the significant result in POD incidence in the subgroup of neuraxial anesthesia. The more studies and larger sample size may be the main cause of this result. The potential reasons above mentioned also need to be further proved in the updated meta-analysis with increasing number of high-quality studies in this field.

There are several limitations in this meta-analysis. Firstly, over 90% of patients are from retrospective studies, which may result in unreliable outcomes due to the selection and recall biases and data loss. Secondly, emergency surgery has been identified as a risk factor of POD (57), however, most of the included trials with patients undergoing both urgent and selective operations did not provide the specific number of urgent and selective patients, which may impact the results. Thirdly, most of the included studies did not clarify whether sedative drugs were used during surgery in the RA group, which might be another factor in interfering with the results. Fourthly, the type and/or dose of general anesthetics varied in the GA group among the included trials, which also is a cause of the uncertain results.

## Conclusion

In this systematic review and meta-analysis, we did not confirm that GA was associated with a higher incidence of POD in surgical patients when compared with RA. The pooled result should be updated by cumulative high-quality studies in the future.

## Data Availability Statement

The original contributions presented in the study are included in the article/Supplementary Material, further inquiries can be directed to the corresponding author/s.

## Author Contributions

XZ and MY independently performed the screening process for titles and abstracts and were responsible for extracting the data. HW and JM performed the screening process for full texts. LZ and HW independently assessed the quality of included studies. HW, LZ, and FY were responsible for adjusting data discrepancies. HW conducted the statistical analysis and made the figures and tables. XZ prepared the manuscript. FY and ZW supervised the whole process and ensured the effectiveness of the meta-analysis. All authors have read and approved the submission of the final manuscript.

## Funding

This work was funded by the National Natural Science Foundation of China (Grant No. 81760257).

## Conflict of Interest

The authors declare that the research was conducted in the absence of any commercial or financial relationships that could be construed as a potential conflict of interest.

## Publisher's Note

All claims expressed in this article are solely those of the authors and do not necessarily represent those of their affiliated organizations, or those of the publisher, the editors and the reviewers. Any product that may be evaluated in this article, or claim that may be made by its manufacturer, is not guaranteed or endorsed by the publisher.

## Supplementary Material

Supplementary Figure 1 Risk of bias graph: review authors' judgements about each risk of bias item presented as percentages across all included studies.

Supplementary Figure 2 Risk of bias summary: review authors' judgements about each risk of bias item for each included study.

Supplementary Table 1 The guidelines of the 2009 PRISMA (Preferred Reporting Items for Systematic reviews and Meta-analyses).

Supplementary Table 2 Bias risk of observational studies (retrospective and prospective) by the Newcastle-Ottawa Quality Assessment Scale.
