# Supplementary figures and images for: The Effect of General Anesthesia vs. Regional Anesthesia on Postoperative Delirium—A Systematic Review and Meta-Analysis
Source: Front Med (Lausanne). 2022 Mar 28;9:844371. doi: 10.3389/fmed.2022.844371 (PMC8995788; doi:10.3389/fmed.2022.844371)

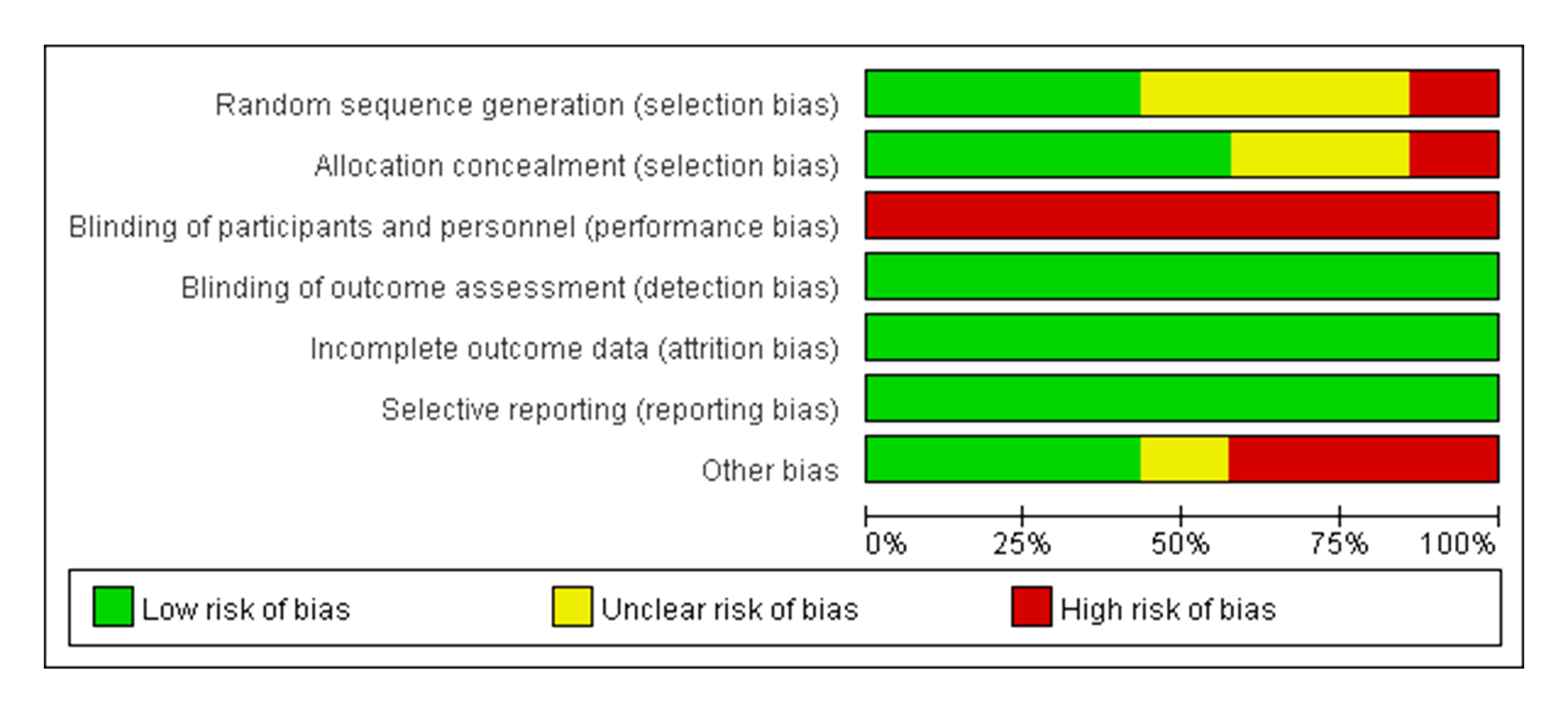

Supplement: Supplementary Figure 1 — Risk of bias graph: review authors' judgements about each risk of bias item presented as percentages across all included studies. [file Image_1.TIF]

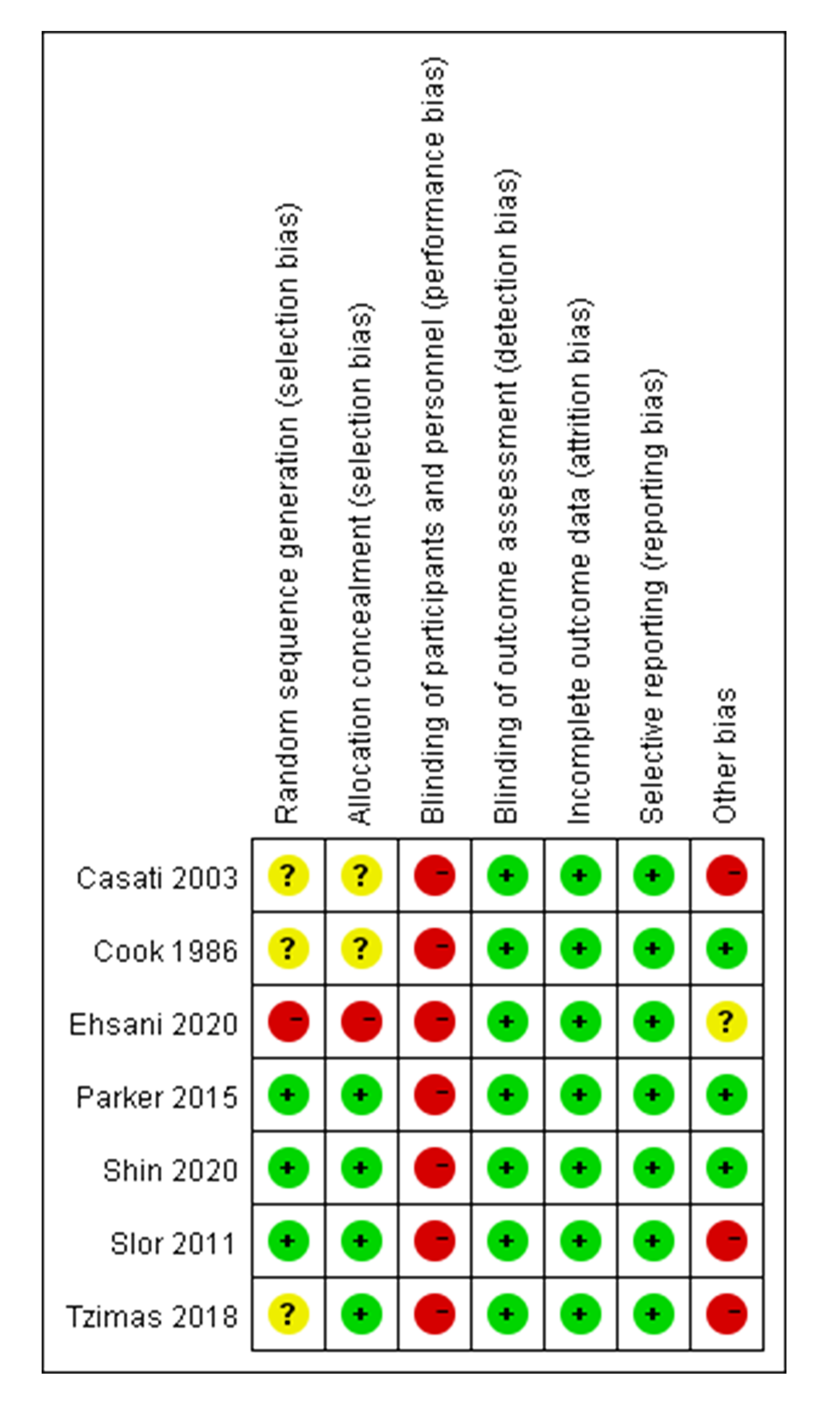

Supplement: Supplementary Figure 2 — Risk of bias summary: review authors' judgements about each risk of bias item for each included study. [file Image_2.TIF]
