# Supplementary material for: The Effect of General Anesthesia vs. Regional Anesthesia on Postoperative Delirium—A Systematic Review and Meta-Analysis
Source: Front Med (Lausanne). 2022 Mar 28;9:844371. doi: 10.3389/fmed.2022.844371 (PMC8995788; doi:10.3389/fmed.2022.844371)
Supplement: Supplementary Table 1 — The guidelines of the 2009 PRISMA (Preferred Reporting Items for Systematic reviews and Meta-analyses). [file Table_1.DOC]

| **Section/topic** | **#** | **Checklist item** | **Reported on page #** |
| --- | --- | --- | --- |
| **TITLE** | | |  |
| Title | 1 | The effect of general anesthesia versus regional anesthesia on postoperative delirium--A systematic review and meta-analysis | P 1 |
| **ABSTRACT** | | |  |
| Structured summary | 2 | **Background:** Postoperative delirium (POD) commonly occurs in patients following major surgeries, and is associated with adverse prognosis. The modes of anesthesia may be associated with POD occurrence. General anesthesia (GA) causes the patient loss of consciousness by altering the levels of some neurotransmitters as well as signaling pathways. We conducted this meta-analysis to investigate the effect of GA versus regional anesthesia (RA) on POD incidence in surgical patients.  **Methods:** The databases of Pubmed, Embase and Cochrane Library were searched till 22nd Oct 2021. The eligible criteria were participants aged 18 years or older, patients undergoing surgery under GA and RA; and articles reporting the effect of GA versus RA on POD incidence. RevMan 5.3 was used to perform statistical analyses.  **Results:** 21 relevant trials with a total of 1702151 patients were included. The pooled result using random-effects model with OR demonstrated significant difference in POD incidence between patients with GA and RA (OR = 1.15, 95% CI: [1.02, 1.31], I^2^ =83%, *p* for effect = 0.02). We did not obtain the consistent pooled result after sensitivity analysis (OR = 0.95, 95% CI: [0.83, 1.08], I^2^ =13%, *p* for effect = 0.44) and excluded the articles without the information on preoperative cognitive or neuropsychological assessmen (OR = 1.12, 95% CI: [1.00, 1.25], I^2^ =80%, *p* for effect = 0.05), respectively.  **Conclusion:** This meta-analysis could not identify that GA was significantly associated with POD occurrence in the surgical patients compared with RA. | P 2 |
| **INTRODUCTION** | | |  |
| Rationale | 3 | According to reports, POD accounts for 11%-51% of patients after major surgery, and is independently associated with prolonged intensive care unit (ICU) and hospital stay, long-term postoperative cognitive dysfunction, and increased mortality. GA is the anesthesia method that keeps the patient in the state of unconsciousness, analgesia and relaxed skeletal muscle through intravenous and/or inhaled general anesthetics during surgery. Some anesthetics act on the central nervous system to produce the effects of consciousness loss and analgesia by altering the levels of some neurotransmitters as well as signaling pathways. However, the neuraxial anesthesia and PNB can contribute to analgesia and skeletal muscle relaxation without affecting the consciousness of patient. Therefore, the patients undergoing GA are theoretically more likely to develop POD. Unexpectedly, the results of anesthesia mode associated with POD incidence are not consistent based on previous studies. | P 2 |
| Objectives | 4 | We performed this systematic review and meta-analysis to clarify the effect of GA versus RA on the incidence of delirium in adult surgery patients. | P 3 |
| **METHODS** | | |  |
| Protocol and registration | 5 | No registration |  |
| Eligibility criteria | 6 | The inclusion criteria were 1) participants aged 18 years or older; 2) patients undergoing surgery under general and regional or local anesthesia; and 3) articles reporting the effect of GA versus RA on POD incidence. The exclusion criteria were: 1) duplicate articles; 2) participants younger than 18 years old; 3) review or meta-analysis; 4) articles published as an abstract, letter, case report, basic research, editorial, note, method, or protocol; 5) articles presented in a non-English language; 6) studies without a specific number of patients with and/or without delirium; and 7) studies of all patients receiving GA or RA. | P 3 |
| Information sources | 7 | We searched the database of Pubmed, Embase and Cochrane Library using the PICOS (Population, Intervention, Comparison, Outcome, Study design) methods by the time of Oct 22, 2021. | P 3 |
| Search | 8 | The entry terms included “general anesthesia” OR “general anaesthesia” AND “local anesthesia” OR “local anaesthesia” OR “regional anesthesia” OR “regional anaesthesia” OR “spinal anesthesia” OR “spinal anaesthesia” OR “epidural anesthesia” OR “epidural anaesthesia” OR “neuraxial anesthesia” OR “neuraxial anaesthesia” AND “delirium” OR “confusion” OR “agitation” OR “acute confusional state” OR “acute confusional syndrome”, and the search field was “title and abstract.” Because we sought to investigate all studies about the effect of GA versus RA on POD incidence in adult patients undergoing surgery, we did not constrain the search terms for study designs. | P 3 |
| Study selection | 9 | The inclusion criteria were 1) participants aged 18 years or older; 2) patients undergoing surgery under general and regional or local anesthesia; and 3) articles reporting the effect of GA versus RA on POD incidence. The exclusion criteria were: 1) duplicate articles; 2) participants younger than 18 years old; 3) review or meta-analysis; 4) articles published as an abstract, letter, case report, basic research, editorial, note, method, or protocol; 5) articles presented in a non-English language; 6) studies without a specific number of patients with and/or without delirium; and 7) studies of all patients receiving GA or RA. | P 3 |
| Data collection process | 10 | Two authors were independently responsible for reviewing the titles, abstracts or both and summarized the data of the included literatures. Another three authors were in charge of the data discrepancy adjustment. | P 4 |
| Data items | 11 | 1) authors; 2) publication year; 3) study designs; 4) country of publication; 5) total number of participants in each study; 6) percentage of males; 7) mean age of all the participants; 8) procedures that the participants underwent; 9) the volatile anesthetic in patients underwent GA; 10) the anesthetic method of RA; 11) number of patients with and without POD; 12) methods of POD assessment; and 13) the follow-up time. | P 4 |
| Risk of bias in individual studies | 12 | Two authors independently assessed the quality of included studies. The Cochrane Collaboration Risk of Bias Assessment tool was used to assess the risk of bias of all included RCTs, and the Newcastle-Otawa Quality Assessment Scale (NOS) was used to assess the bias risk of observational trials. If the two authors had the different assessment results, they consulted the third or the forth one. Eventually, we reached consensus. | P 3 |
| Summary measures | 13 | The dichotomous outcome were reported as odds ratios (OR) with 95% confidence interval (CI). The statistical tests were two-sided and *p* value for overall effect <0.05 was considered significant differences. | P 4 |
| Synthesis of results | 14 | The values of I^2^ and the Mantel-Haenszel chi-square test (*p* value for heterogeneity) were used to evaluate the heterogeneity of included studies. And the values of I^2^<40%, 40%-60%, and >60% represented low, moderate and high heterogeneity, respectively. A *p* value for heterogeneity<0.1 or I^2^ >50% was regarded as high heterogeneity and the method of random-effect model analysis was applied to pool the data. | P 4 |

Page 1 of 2

| **Section/topic** | **#** | **Checklist item** | **Reported on page #** |
| --- | --- | --- | --- |
| Risk of bias across studies | 15 | We used NOS to assess the risk of bias in observational studies (retrospective and prospective), and all 14 trials obtained seven stars or more, indicating high quality (Supplementary Table 2) (17-19, 21, 22, 25-31, 35, 36). We used the Cochrane Collaboration Risk of Bias Assessment tool to assess the risk of bias in RCTs . Due to the considerable procedural difference of GA and neuraxial anesthesia or PNB, it is extremely difficult to conduct blindness in participants. Therefore, the performance bias was high risk in all included RCTs and Non-RCTs. The included studies clearly assessed random sequence generation (three studies-42.9%), allocation concealment (four-studies-57.1%), blinding of participants (0%), blinding of outcome assessment (seven studies-100%), incomplete outcome data (seven studies-100%), and selective outcome reporting (seven studies-100%), and the other bias (three-42.9%) | P5-6 |
| Additional analyses | 16 | We conducted sensitivity analysis to address high heterogeneity (I^2^>50%) through the methods of one-by-one article removal. Lastly, we performed the subgroup analyses according to study designs (retrospective and prospective), male percentage (≥50% and <50%), age gaps (≥80 years, 70-80 years, 60-70 years and <60 years), and anesthesia methods (neuraxial anesthesia and PNB) in RA grouo to observe if these risk factors could affect the outcome. | P4 |
| **RESULTS** | | |  |
| Study selection | 17 | See Figure 1 | P 5 |
| Study characteristics | 18 | For each study, present characteristics for which data were extracted (e.g., study size, PICOS, follow-up period) and provide the citations. | P 5 |
| Risk of bias within studies | 19 | Present data on risk of bias of each study and, if available, any outcome level assessment (see item 12). | P 5-6 |
| Results of individual studies | 20 | For all outcomes considered (benefits or harms), present, for each study: (a) simple summary data for each intervention group (b) effect estimates and confidence intervals, ideally with a forest plot. | P 6 |
| Synthesis of results | 21 | The pooled result using random-effects model with OR demonstrated significant difference in POD incidence between patients with GA and RA (OR = 1.15, 95% CI: [1.02, 1.31], I^2^ =83%, *p* for effect = 0.02) (Figure 2). I^2^ =81% and the funnel plots demonstrated the considerable heterogeneity of included trials (Figure 3A). The sensitivity analysis was performed to solve the high heterogeneity by the method of one-by-one literature removal and found that 6 trials were the main sources of heterogeneity (Figure 3B) (18, 19, 24, 30, 35, 36). We conducted post hoc meta-analysis for the remaining articles using a fixed-effects model with OR, and the pooled result was not consistent with that prior to sensitivity analysis (OR = 0.95, 95% CI: [0.83, 1.08], I^2^ =13%, *p* for effect = 0.44) (Figure 4). Additionally, we excluded the articles without the information on preoperative cognitive or neuropsychological assessment, and did not obtain the statistical difference in POD incidence between patients with GA and RA (OR = 1.12, 95% CI: [1.00, 1.25], I^2^ =80%, *p* for effect = 0.05) (Figure 5). | P 6 |
| Risk of bias across studies | 22 | Present results of any assessment of risk of bias across studies (see Item 15). | P 5-6 |
| Additional analysis | 23 | The subgroup analyses according to study designs, male percentage (≥50% and <50%), mean (or median) age gaps (≥80 years, 70-80 years, 60-70 years and <60 years), and anesthesia methods in RA group (neuraxial anesthesia and PNB) demonstrated the significant difference in retrospective articles (OR = 1.23, 95% CI: [1.08, 1.39], *p* for effect = 0.001) (Figure 6), male percentage<50% (OR = 1.26, 95% CI: [1.09, 1.46], *p* for effect = 0.002) (Figure 7), age gap between 60 and 70 years (OR = 1.20, 95% CI: [1.07, 1.35], *p* for effect = 0.002) (Figure 8), and neuraxial anesthesia (OR = 1.15, 95% CI: [1.01, 1.31], *p* for effect = 0.03) (Figure 9). However, we did not obtain statistical difference in the subgroups in prospective studies (OR = 0.91, 95% CI: [0.55, 1.49], *p* for effect = 0.70) (Figure 6), male percentage≥50% (OR = 0.96, 95% CI: [0.82, 1.12], *p* for effect = 0.60) (Figure 7), age gaps≥80 years (OR = 0.98, 95% CI: [0.61, 1.57], *p* for effect = 0.93), 70-80 years (OR =1.93, 95% CI: [0.66, 5.60], *p* for effect = 0.23), <60 years (OR = 0.67, 95% CI: [0.26, 1.71], *p* for effect = 0.40) (Figure 8), and PNB group (OR = 1.64, 95% CI: [0.70, 3.87], *p* for effect = 0.26) (Figure 9). [see Item 16]). | P 6 |
| **DISCUSSION** | | |  |
| Summary of evidence | 24 | Although this meta-analysis concluded that compared with RA, the incidence of POD significantly increased in GA patients, we did not obtain the positive result after solving the high heterogeneity of included trials and excluded the trials did not provide the information on preoperative cognitive or neuropsychological assessment, respectively. Besides, subgroup analyses showed the statistical difference in retrospective studies, studies with male percentage<50%, studies with mean (or median) age gap 60-70 years, and studies with neuraxial anesthesia group in RA group. However, we did not obtain the considerable difference in POD occurrence between the patients with GA and RA in the trials of prospective designs, the male percentage≥50%, patients with other mean (or median) age gaps except 60-70 years, and patients undergoing PNB in the RA group. | P 6 |
| Limitations | 25 | Firstly, over 90% of patients are from retrospective studies, which may result in unreliable outcome due to the selection and recall biases and data loss. Secondly, emergency surgery has been identified as a risk factor of POD (59), however, most of included trials with patients undergoing both urgent and selective operation did not provide the specific number of urgent and selective patients, which may impact the results. Thirdly, most of included studies did not clarify whether sedative drugs were used during surgery in RA group, which might be another factor in interfering with the results. Fourthly, the type and/or dose of general anesthetics varied in the GA group among the included trials, which also is a cause of uncertain result. | P 8 |
| Conclusions | 26 | In this systematic review and meta-analysis, we do not confirm that compared with RA, GA is associated with higher incidence of POD in surgical patients, and the pooled result should be updated by cumulative high-quality studies in the future. | P 8 |
| **FUNDING** | | |  |
| Funding | 27 | This work was funded by the National Natural Science Foundation of China (Grant No. 81760257). | P 9 |

*From:*  Moher D, Liberati A, Tetzlaff J, Altman DG, The PRISMA Group (2009). Preferred Reporting Items for Systematic Reviews and Meta-Analyses: The PRISMA Statement. PLoS Med 6(7): e1000097. doi:10.1371/journal.pmed1000097

For more information, visit: **www.prisma-statement.org**.

Page 2 of 2
