# Supplementary material for: The Effect of General Anesthesia vs. Regional Anesthesia on Postoperative Delirium—A Systematic Review and Meta-Analysis
Source: Front Med (Lausanne). 2022 Mar 28;9:844371. doi: 10.3389/fmed.2022.844371 (PMC8995788; doi:10.3389/fmed.2022.844371)
Supplement: Supplementary Table 2 — Bias risk of observational studies (retrospective and prospective) by the Newcastle-Ottawa Quality Assessment Scale. [file Table_2.docx]

Supplementary Table 2. Bias risk of observational studies (retrospective and prospective) by the Newcastle-Ottawa Quality Assessment Scale

| **Study** | **Selection** | | | | **Comparability** | **Outcome** | | | **Total score** |
| --- | --- | --- | --- | --- | --- | --- | --- | --- | --- |
|  | **Representativenes of exposed cohort** | **Selection of unexposed**  **cohort** | **Ascertainment of exposure** | **Outcome of interest** |  | **Assessment of outcome** | **Follow-up long enough for outcomes to occur** | **Adequacy**  **of follow-up** |  |
| Abe 2020 | * | * | * | * | * | * | * | * | 8 |
| Ahn 2019 | * | * | * | * | * |  | * | * | 7 |
| Bilge 2015 | * | * | * | * | * |  | * | * | 7 |
| Chew 2021 | * | * | * | * | * | * | * | * | 8 |
| Choi 2020 | * | * | * | * | * | * | * | * | 8 |
| Ellard 2014 | * | * | * | * | * | * | * | * | 8 |
| Ilango 2016 | * | * | * | * | * | * | * | * | 8 |
| Krenk 2012 | * | * | * | * | * | * | * | * | 8 |
| Li 2019 | * | * | * | * | * |  | * | * | 7 |
| Liu 2014 | * | * | * | * | * |  | * | * | 7 |
| Memtsoudis 2019 | * | * | * | * | * | * | * | * | 8 |
| Nawi 2021 | * | * | * | * | * |  | * | * | 7 |
| Song 2019 | * | * | * | * | * |  | * | * | 7 |
| Song 2021 | * | * | * | * | * | * | * | * | 8 |
